# Bee Tracker—an open‐source machine learning‐based video analysis software for the assessment of nesting and foraging performance of cavity‐nesting solitary bees

**DOI:** 10.1002/ece3.8575

**Published:** 2022-03-07

**Authors:** Anina C. Knauer, Johannes Gallmann, Matthias Albrecht

**Affiliations:** ^1^ Agroscope, Agroecology and Environment Zürich Switzerland; ^2^ Ubique Innovations AG Zürich Switzerland

**Keywords:** behavior, fitness, *Osmia bicornis*, risk assessment, sublethal effects

## Abstract

The foraging and nesting performance of bees can provide important information on bee health and is of interest for risk and impact assessment of environmental stressors. While radiofrequency identification (RFID) technology is an efficient tool increasingly used for the collection of behavioral data in social bee species such as honeybees, behavioral studies on solitary bees still largely depend on direct observations, which is very time‐consuming. Here, we present a novel automated methodological approach of individually and simultaneously tracking and analyzing foraging and nesting behavior of numerous cavity‐nesting solitary bees. The approach consists of monitoring nesting units by video recording and automated analysis of videos by machine learning‐based software. This *Bee Tracker* software consists of four trained deep learning networks to detect bees that enter or leave their nest and to recognize individual IDs on the bees’ thorax and the IDs of their nests according to their positions in the nesting unit. The software is able to identify each nest of each individual nesting bee, which permits to measure individual‐based measures of reproductive success. Moreover, the software quantifies the number of cavities a female enters until it finds its nest as a proxy of nest recognition, and it provides information on the number and duration of foraging trips. By training the software on 8 videos recording 24 nesting females per video, the software achieved a precision of 96% correct measurements of these parameters. The software could be adapted to various experimental setups by training it according to a set of videos. The presented method allows to efficiently collect large amounts of data on cavity‐nesting solitary bee species and represents a promising new tool for the monitoring and assessment of behavior and reproductive success under laboratory, semi‐field, and field conditions.

## Graphical abstract

The foraging and nesting performance of bees can provide important information on bee health and is of interest for risk and impact assessment of environmental stressors. Here, we present a novel video analysis software for the analysis of foraging and nesting behavior of cavity‐nesting solitary bees. The software could be adapted to various experimental setups and allows to efficiently collect large amounts of data on cavity‐nesting solitary bee species under laboratory, semi‐field, and field conditions.

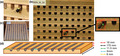

## 1 INTRODUCTION

Bees provide pollination services to wild plants and crops and are essential for biodiversity and human food supply (Garibaldi et al., 2014; Klein et al., 2007; Ollerton et al., 2011). They include important flagship and indicator species and are used for the monitoring and impact assessment of environmental stressors such as habitat degradation, pesticide exposure, or pathogens (Potts et al., 2010, 2016; Schönfelder & Bogner, 2017; Woodard et al., 2020). An important component in the evaluation of bee health is the assessment of reproductive success and foraging behavior, as key drivers of population development and provisioning of pollination services (Artz & Pitts‐Singer, 2015; Ganser et al., 2020; Henry et al., 2012; Siviter et al., 2021). Such assessments require, however, accurate and efficient tools to collect the often large amount of data required to assess bee health, especially if data on individual bees shall be collected (Crall et al., 2018; Nunes‐Silva et al., 2019).

Recent research and environmental risk assessments have mainly focused on the honeybee, *Apis mellifera*, and a few other social bee species (e.g., *Bombus terrestris)* as indicator species (Goulson et al., 2015; Potts et al., 2016). Only relatively recently, there is increased recognition of the fact that the effect of different environmental drivers can substantially vary between bee species and depend on their functional and life‐history traits such as sociality, body size, foraging, or nesting traits (Brittain & Potts, 2011; Sgolastra et al., 2019). While social bee species may compensate for temporary limited stress exposure (e.g., pesticide applications) at a later point in time (Straub et al., 2015), it should directly impair reproductive output in solitary bees (Sgolastra et al., 2019). Risk assessments therefore increasingly consider also solitary bee species for the monitoring of impacts of stressors on bee pollinators, prominently including cavity‐nesting species (Boff et al., 2020; Rundlöf et al., 2015; Stuligross & Williams, 2020; Zurbuchen et al., 2010). In Europe for example, the European Food Safety Authority (EFSA) has proposed to integrate two cavity‐nesting solitary bee species, *Osmia bicornis* and *O. cornuta* for risk assessment of plant protection products on bees, including higher‐tier assessments of sublethal effects on reproductive success (EFSA, 2013; Franke et al., 2021).

**FIGURE 1:**
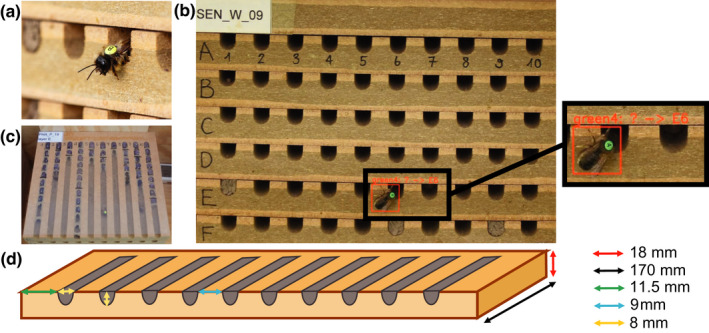
(a) Nesting *Osmia bicornis* female bee marked with an ID tag (unique color–digit combination) attached to its thorax, (b) nesting unit composed of layers (wooden boards) with 10 cavities each, (c) layer with cavities covered with plastic foil for which nesting progress and offspring production can be tracked, and (d) specifications of layers used for nesting units—black: 170 mm; red: 18 mm; green: 11.5 mm; blue: 9 mm; and yellow: 8 mm

Solitary bees can respond through changes in their nesting and foraging behavior to various environmental stressors as pesticides, habitat degradation, or pathogens (Artz & Pitts‐Singer, 2015; Boff et al., 2020; Klaus et al., 2021; Klinger et al., 2021; Siviter et al., 2021; Stuligross & Williams, 2020). However, while foraging behavior of individuals of social bee species such as *A. mellifera* can automatically be recorded with RFID technology (Nunes‐Silva et al., 2019), no such tool is, to our knowledge, currently available for the collection of such data for solitary bees. As studies with cavity‐nesting solitary bees typically require nesting units with numerous scattered nesting cavities (Figure 1), RFID, which has a short reach of the signal (Nunes‐Silva et al., 2019), is difficult to implement. Furthermore, tracking foraging behavior and reproductive success of multiple individual females requires correct identification and assignment of the cavities used for nesting by individual females, which can only be achieved with a large number of readers at high costs. So far, studies on solitary bee species have therefore largely depended on direct visual observation to monitor foraging behavior or the nesting progress of individual females (Artz & Pitts‐Singer, 2015; Franke et al., 2021), which is very time‐consuming, hampering research and environmental risk assessment with solitary bee species.

Software can be used to automatically detect animals in images or analyze animal behavior recorded with videos (Eikelboom et al., 2019; Pennington et al., 2019). For bees, automated image classification was used to count nests of ground‐nesting solitary bees (Hart & Huang, 2014), to monitor the activity of individually tagged honeybees (Chen et al., 2012; Odemer, 2021) and for the detection of parasites and pollen‐bearing in honeybees (Rodriguez et al., 2018; Schurischuster et al., 2018). Here, we present new machine learning‐based software, which can automatically extract and analyze data on the foraging and nesting behavior of individually marked, cavity‐nesting solitary bees from videos. The software is provided free and open‐source including the underlying Python code, as well as a user manual, which makes the software also accessible to users who have no programming background. The machine‐learning networks that permit to train the software and parameters of the input file can be adapted to specific requirements, which allows to use the software in a wide range of experimental setups.

## 2 METHODS

### 2.1 Bee Tracker software

The *Bee Tracker* software is able to recognize bees entering and leaving cavities at a nesting unit. Individual bees can be identified if they are marked with ID tags from marking kits conventionally used for honeybee queen rearing (Figure 1). In the published open‐source version of the software, digits from 1 to 8 and the colors white, yellow, and green (up to 24 unique digit–color combinations) can be recognized. Moreover, the software can identify each nesting cavity of nesting units (constructed as in Figure 1). Cavities get an ID based on their position in the nesting unit (according to its “row” and “column” in the nesting unit, see the manual provided in the Supporting Information for further details). In the published version of the software, cavities of up to 12 rows and 10 columns (up to 120 cavities) per nesting unit can be identified. The software is further capable to detect and measure the entering and leaving of a cavity by an individual bee and the video timestamp of each of these events. From the collected list of events and some set input parameters (see below), the software can assign females to the cavity they are nesting in, calculate flight duration, and count the number of cavities a bee probes until it finds the one it is nesting in (nest recognition; see Artz & Pitts‐Singer, 2015).

Before the software can be used for the collection of this data, the precision of the software needs to be evaluated for the setup in use and, if unsatisfactory, the software must be trained on a set of representative videos. The machine‐learning network (see below: *Machine‐learning algorithms and training of models*) can further be used to expand the spectra of bee and cavity IDs that the software is able to recognize. How the software can be trained to the setup in use and/or additional bee and cavity IDs is described in the manual (provided in the Supporting Information).

### 2.2 Input videos

The input videos must be in MP4 format and have an aspect ratio of 16:9. The software was developed and validated with an aspect ratio of 3860 × 2160 (4K), which returns well‐resolved images that generate outputs with a high measurement precision. A lower resolution could impair the precision, but the software can still process the input.

### 2.3 Generated output

The software will create a new subfolder within the selected result folder for each input video. Inside each subfolder, the following outputs are stored by the software:

1. **all_events_unfiltered:** Inside this csv file, all detected events are listed containing the video timestamp, the bee ID, the event type (entering or leaving), and the cavity ID. This list is completely unfiltered and may contain errors.
2. **error_corrected_events:** This csv file contains all events that remain after error correction: Events with unidentified bee IDs get removed. The software additionally identifies missing events within sequences of enter–leave–enter. Such sequences with missing events are not considered for the creation of below‐described output files (address_book, nest_recognition, flight_list). Besides the video timestamp, the bee ID, the cavity ID, and the type of event (entering or leaving a cavity), this file therefore also indicates for each event whether it was used for the output files address_book, nest_recognition, and flight_list.
3. **address_book:** This csv file contains all bees that were assigned to a nest and lists the according bee and cavity IDs. These data (assignments between individual bees and the cavity (or cavities, respectively) they are nesting in) are of interest for assessments of nesting progress and reproductive success of individual nesting females. In order to assign females to cavities that are used for nesting (in contrast to simply probed cavities not used for nesting), a cavity is only assigned to an individual bee if (i) the bee stays inside the cavity for a time span that is minimally required by a nesting bee to unload collected pollen for offspring provision, and (ii) the bee does not enter another cavity during a time span that is minimally required by a bee to collect pollen or material such as mud for nest construction (e.g., construction of brood cell walls). The default setting of these two time spans is both 40 s in the published open‐source version of the software. These values were chosen based on over 20 h of direct observation of *Osmia bicornis* females nesting in a natural habitat in Switzerland (Bättig D., unpublished data). However, the species under study or the experimental setting may require adjustment of these threshold values. This can be done in the “config” file of the software, which can be selected as an optional input file for the analysis (see software manual in the Supporting Information).
  Nesting progress, that is, the number of produced brood cells and offspring, can be tracked by repeatedly photographing the nest cavities (Figure 1), for example, before and after an assessment day. Linking these data with the *address_book* file (created from a video recorded on the same assessment day) based on cavity IDs permits to measure individual reproductive success per female for this time period.
4. **nest_recognition:** This csv file contains the number of cavities a female enters before finding its nest (i.e., number of probed “wrong” cavities before finding the “correct” nesting cavity). Besides the bee ID and the number of probed cavities, the file also lists the video timestamp.
5. **flight_list:** This csv file provides flight durations of individual females from leaving the nesting cavity until returning to it again (i.e., foraging trip or mud collection duration). Besides the bee ID and the flight duration, the file also lists the video timestamp. If of interest, flight activity, defined here as females that perform at least one flight during the observation time, can be assessed by classifying females that are listed in the flight_list file as active. For this measurement, the number of total, alive females needs to be known, however, which can be assessed by taking pictures of the nest layer (Figure 1) during the night when females are roosting inside cavities.
6. **Visualization:** Through the “visualize results” option, a video file in mp4 format can be created with all detected events visualized. This file can be used to manually check the performance of the software and to find potential errors, which can be used to retrain the software (see below) and improve the precision.

### 2.4 Measurement of precision

To measure the precision of the software in a set of videos, the created visualization videos can be manually checked and the correct assignment of the bee ID, cavity ID, and event type (entering or leaving) can be reviewed for single events. Only events listed in the error_corrected_events file as events that were used to create measurements in output files (last column contains a yes) should be checked and used for the measurement of precision. Precision can be calculated as the proportion of fully correct assignments as *Precision* = *TP* / (*TP* + *FP*), where TP is the number of true positives and FP the number of false positives. The measured precision is valid for all extracted measurements: assignment of females to their nest cavity, flight duration, and number of probed cavities.

The software was designed to achieve a high precision at the expense of the recall (fraction of events that was retrieved), which is of minor interest in this type of analysis as it only affects the sample sizes but not the extracted measurements themselves. We therefore did not implement the possibility to assess the recall.

### 2.5 Machine‐learning algorithms and training of models

The *Bee Tracker* software uses a combination of three machine‐learning algorithms to generate the above‐mentioned outputs: the Faster R‐CNN object detection pipeline (Ren et al., 2017), a YoloV3 (Redmon & Farhadi, 2018) object detection network, and a custom Keras image classification network (Chollet, 2015). The software takes a video of a nesting unit as described above as input and as a first step detects all marked bees and cavities in each video frame using two trained Faster R‐CNN networks. Subsequently, the marker tags (unique digit–color combination; Figure 1) are identified by a YoloV3 network on each previously detected bee. Additionally, all identified markers are further classified into the digits 1–8 by a custom Keras network. Knowing the cavity positions and bee positions alongside with the bee ID for each individual frame, a custom object tracking algorithm is applied to these data in order to link the individual frames together and obtain a movement path for each bee. By analyzing the start and end point of each detected movement path, the software is able to detect cavity entering and leaving events.

The software relies on the four previously mentioned trained machine‐learning models. The model for detecting the bees was trained on 1303 individual images. The cavity detection model was trained on 120 individual images of nesting units; each nesting unit contained between 60 and 130 cavities. The color tag detection model was trained on 4921 individual images of bees, and the digit classification model was trained with 10,347 individual images of number tags. Additionally, various data augmentation techniques were applied such as rotations, random brightness adjustments, random contrast adjustments, and random saturation. Further detailed information about the model trainings is provided in the software manual (see Supporting Information).

### 2.6 Software evaluation

To evaluate the software and measure the precision of the analyses and generated outputs, we recorded a total of 23 videos from 15 nesting units during two consecutive days using the nesting units as described in Figure 1. All nesting units were placed in large flight cages (54 m^2^) that contained sufficient floral sources for offspring provisioning by nesting female *Osmia bicornis* (sown purple tansy, buckwheat, and/or field mustard). A total of 24 females marked with the above‐described 24 unique digit–color ID tags were released into each of these flight cages, and videos were recorded after initiation of nesting. Each video was recorded between 9 a.m. and 3 p.m. when flight activity was high; recording times ranged between 2 and 4 h. Cameras were placed at a distance of 1 m from the nesting unit with frontal view (camera placed at same height as nesting unit). From the recorded videos, 8 randomly selected ones were used to train the software to this experimental setup, while the remaining 15 videos were used to measure precision. Precision was assessed by manually checking 180 randomly selected events (12 events per video) for their correctness using the *visualization* option of the software (see above). Only events that were used for the generation of output csv files (after error correction) were inspected as described above.

For the comparability of bee health under different environmental conditions (e.g., different field sites with variable habitat quality or flight cages with/without pesticide application), a similar precision across videos is required. We therefore tested whether precision varied between videos in our set of evaluated videos in a generalized linear model with a binomial distribution. The correctness of the detected event (correct or wrong) was included as the response variable and the video ID as explanatory variable. As the software only assigns females to a nest that are active during recording and fulfill certain criteria (as described in the section address_book), we further fitted a generalized linear model with a binomial distribution to test whether the proportion of females that can be assigned to a nest cavity per video depends on the video recording time. The analysis was done in R 4.1. (R Core Team, 2021).

## 3 RESULTS

On average, the *Bee Tracker* software could successfully assign 67% (lower CI: 61%, upper CI: 71%) of the alive females to a nest per video. The probability that a female gets assigned to a nest did not depend on video recording time (χ^2^ = 1.82, *p* = .18), which ranged between 2 and 4 h. Per video, the software generated on average nest_recognition files with 80 measures of nest recognition (the number a female enters any cavity until it finds the one it is nesting in) per hour and flight_list files with 61 flights per hour.

**FIGURE 2:**
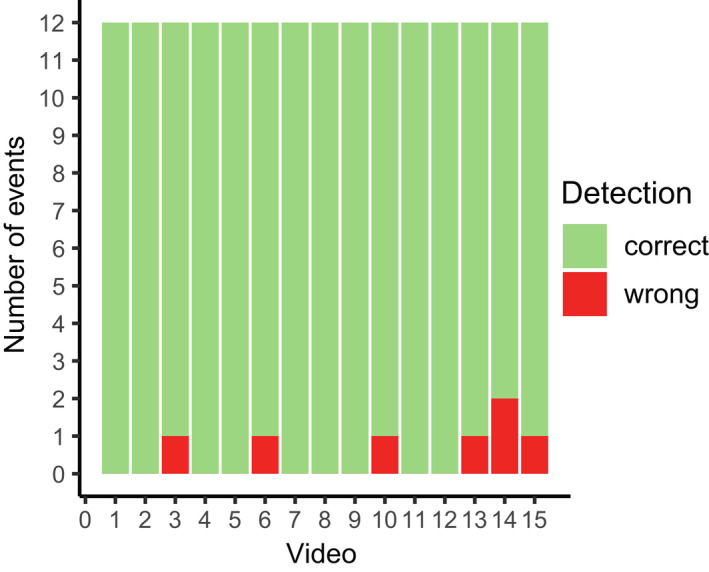
Number of events that were detected correctly or with an error by the *Bee Tracker* software in the 15 videos that were checked manually

The precision of the software was evaluated by manually checking the created visualization videos and reviewing the correct assignment of the bee ID, cavity ID, and event type (entering or leaving) of single events. Per video, 96.1% (lower CI: 92.6%, upper CI: 98.3%) of the checked events were detected correctly on average, whereas precision did not depend on the video that was used for the analysis (χ^2^ = 13.95, *p* = .45; Figure 2). The seven errors that were found all related to the bee ID. Three errors were caused by a wrong color detection: Green was classified falsely as yellow in all these cases. The remaining four errors were caused by an ID swap between two bees that had crossing movement paths, which led to a commutation of the IDs between bees.

## 4 DISCUSSION

### 4.1 Software performance

The *Bee Tracker* software is a helpful tool to collect large amounts of data on the nesting and foraging behavior of cavity‐nesting solitary bees in an automated way. It identifies individual nesting females and assigns them to their nests. This permits to obtain robust data on per female reproductive success, if nesting progress within nests is additionally recorded. Moreover, the software counts the number of cavities a female probes until it finds its nest, collects information on the flight duration, and allows to assess flight activity. Once the software is trained for the experimental setup in use, the method requires low labor input but can generate large data sets with a high measurement precision. Here, we showed that a precision of 96% can be achieved with a relatively low training effort of about 30 working hours. Minor adaptations may further improve the performance of the software.

The precision of the *Bee Tracker* exceeds precision values typically found in automated image analysis software (Eikelboom et al., 2019; Gallmann et al., 2020), but reaches values typical for bee counters (Odemer, 2021). The software may, however, only achieve the here reported precision of 96% in experiments with a similar setup, with respect to light conditions during video recording, hues and digits of bee IDs, and the shape, size, and location of the nest cavities in the nesting units. For variant setups, the training of the software may need to be repeated to achieve a comparable measurement precision of the software analysis. While errors by bee ID swapping cannot be entirely avoided due to the limitations of the centroid object tracking algorithm used by the software, errors caused by color misclassifications between green and yellow were probably caused by the convergence of spectra under different light conditions and could likely be reduced by choosing colors for ID tags with more distinct spectra. Thus, while an increased training effort may reduce the error rate, replacing either green or yellow by, for example, blue or red ID tags may completely eliminate color misclassifications, which would increase the precision to 98% in our data set.

A main advantage of the *Bee Tracker* is the large data sets that can simultaneously be collected with relatively low time and labor input. Direct observations of the nesting activity of individually marked bees, in comparison, are very challenging and nearly impossible in experimental setups with large individual numbers and several sites (or plots/cages), where bees needed to be observed simultaneously. Researchers therefore used videos for the assessments of individual behavior in solitary bees (McKinney & Park, 2012), which are very time‐consuming to manually evaluate. Despite this advantage of the *Bee Tracker*, the method also has some limitations. The use of the software is restricted to relatively large bee species that allow fixing ID tags on the bees’ thorax. Furthermore, the current version of the *Bee Tracker* software was trained on the model bee species *Osmia bicornis*. Although bee recognition and the classification of movement (entering or leaving a cavity) seemed to work equally precise when tested on the closely related species *O. cornuta* (Knauer A., personal observation), further training may be required when working with other solitary bee species to obtain full precision of the software. Furthermore, the current version of the software can only analyze the above‐described 24 unique color–digit‐based bee IDs and identify cavities with a certain size and shape that are arranged in the nesting unit as described (Figure 1). These limits can, however, be adapted by training the software to additional bee IDs (with more digits or colors) and different nesting units. In field studies of natural populations, where bees cannot be tagged after hatching (and before the release into flight cages), nesting units as the ones described in Figure 1 with nest cavities open at the top can be used to capture bees from the cavities during inactivity (e.g., during rain or at night when bees are usually roosting inside cavities) for tagging. The software could therefore be used in various experimental setups to study the behavior of solitary, cavity‐nesting bees that can be established in standardized nesting units.

### 4.2 Research purpose

The effect of different stressors can vary between species and depend on their functional traits such as body size, sociality, or mode of nesting (Brittain & Potts, 2011; Sgolastra et al., 2019). A range of solitary bee species are therefore increasingly studied for the assessment and monitoring of stressors on pollinators (Boff et al., 2020; Ganser et al., 2020; Klaus et al., 2021; Stuligross & Williams, 2020; Zurbuchen et al., 2010). The *Bee Tracker* software can be a helpful tool to efficiently collect robust data on individual nesting and foraging behavior of cavity‐nesting solitary bees.

The assessment of foraging behavior can be a relevant addition to the direct measurement of fitness in bees. In social bee species, the number of adult bees, brood cells, and the amount of food stores (honey and pollen) are used as indicators of colony strength and vitality (Dainat et al., 2020; Hernandez et al., 2020). RFID technology has furthermore been used for the monitoring of foraging behavior in social species as it can perform individual bee recognition and detect the inbound and outbound movements of tagged bees at the nest entrance where the antenna and reader are placed (Nunes‐Silva et al., 2019). With this technology, flight activity, homing ability, and flight duration of social bees can be studied (Henry et al., 2012; Schneider et al., 2012; Stanley et al., 2016; Tenczar et al., 2014). In solitary bees, reproductive success, measured by brood cell or offspring production, is the most important proxy of fitness (Rundlöf et al., 2015; Stuligross & Williams, 2020; Zurbuchen et al., 2010). The *Bee Tracker* software can furthermore be used to measure reproductive output for individual nesting females and to collect large amounts of behavioral data to supplement and better understand measurements of reproductive success and fitness in solitary, cavity‐nesting bees.

Behavioral data can contribute to the understanding of behavior‐mediated impacts of environmental stressors on reproduction of solitary bee species (Artz & Pitts‐Singer, 2015). Pesticide exposure, for example, can impair orientation and memory (Siviter et al., 2018) and cause a reduction in nest recognition or foraging activity (Artz & Pitts‐Singer, 2015; Franke et al., 2021). Flight duration may also be increased by habitat degradation or food competition, which can cause increased flight distances to food sources (Gathmann & Tscharntke, 2002). Pathogens can reduce homing ability in honeybees (Li et al., 2013) or cause a premature onset of foraging and reduce the total activity span of foragers (Benaets et al., 2017). Overall, understanding bees’ foraging and flight activities can provide valuable information for evaluating the impact of a wide range of environmental stressors on bees. For example, behavioral data collected with RFID contributed to the detection of sublethal adverse effects of neonicotinoids, which finally led to the ban of several compounds from this class of insecticides in the European Union (Gross, 2013).

## 5 CONCLUSION

The *Bee Tracker* software is an efficient tool to collect large amounts of data on foraging and nesting behavior of cavity‐nesting solitary bee species. We hope it will contribute to a more accurate and in‐depth study of these behavioral aspects and to an increased consideration of solitary species for the monitoring of impacts of stressors on bees. Such monitoring is essential for the protection of wild pollinators and the vital pollination services they provide to wild plants and crops.

## CONFLICT OF INTEREST

The authors declare no conflict of interest.

## AUTHOR CONTRIBUTION

**Anina Catharina Knauer:** Conceptualization (equal); Data curation (lead); Funding acquisition (equal); Methodology (equal); Project administration (lead); Visualization (equal); Writing – original draft (lead). **Johannes Gallmann:** Conceptualization (equal); Methodology (equal); Software (lead); Visualization (equal); Writing – original draft (supporting). **Matthias Albrecht:** Conceptualization (equal); Funding acquisition (equal); Supervision (lead); Writing – original draft (supporting).

## Supporting information

Supplementary Material

## Data Availability

Data and the software including the underlying Python code are available in Dryad (https://doi.org/10.5061/dryad.08kprr546).
