# Supplementary material for: Bee Tracker—an open‐source machine learning‐based video analysis software for the assessment of nesting and foraging performance of cavity‐nesting solitary bees
Source: Ecol Evol. 2022 Mar 7;12(3):e8575. doi: 10.1002/ece3.8575 (PMC8928898; doi:10.1002/ece3.8575)

Bee Tracker Manual

1 REQUIREMENTS

In order to be able to run the Software, your Windows computer should have an Nvidia GPU with at least 8 GB of RAM.

2 INSTALLATION

1. Unzip the *BeeTracker.zip* file and place it on any location on your computer.
2. Optionally, make a shortcut of the *BeeTracker.exe* which can be found inside the *BeeTracker* folder and place the shortcut on your Desktop or any other location.
3. Double click the *BeeTracker.exe* shortcut to start the program.

3 Quick start

1. Start the Bee Tracker software. It should look like the screenshot on the right.
2. Select one or multiple input videos either by clicking on “Select Input Videos” or by directly typing the path(s) into the window. More than one video can be analyzed simultanously by separating the paths with ”;”.
3. Select a results folder. All results, including intermediate results, will be saved to this results folder.
4. Optionally, a *config* file can be provided. See the instructions below for further information.
5. Choose whether you want to visualize the results or not. If you activate this option, the software will output a visualization video to the results folder.
6. Click “Start” to start the computation.

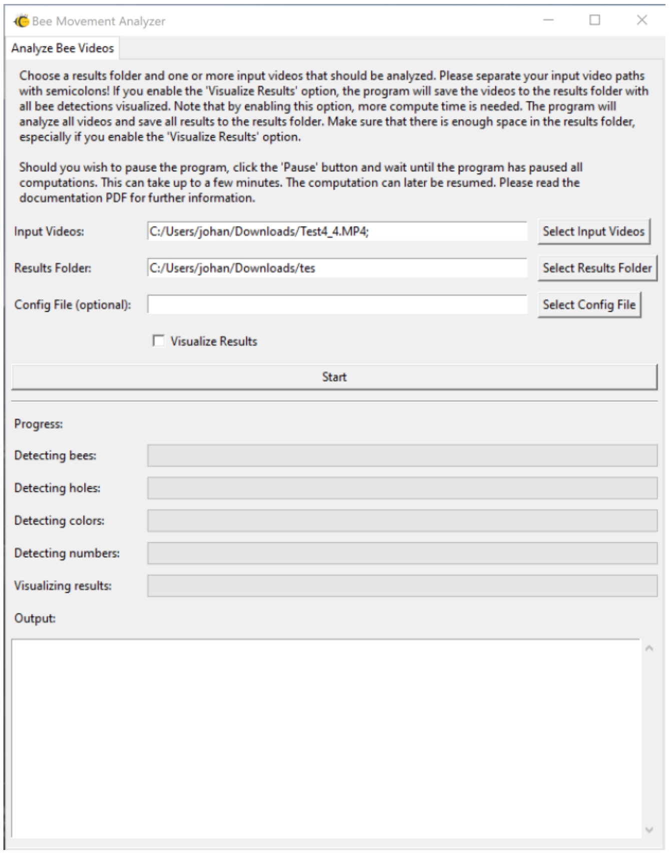

4 DETAILED SOFTWARE DESCRIPTION

4.1 Overview

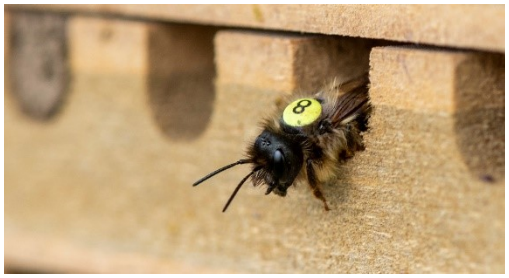

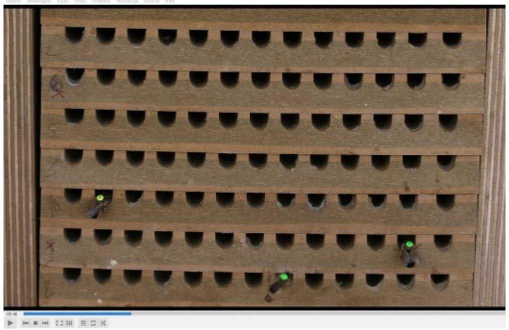

The Bee Tracker Software is able recognize positions of cavities in the nesting unit and bee IDs composed of a color-digit combination on the thorax of the bees (see image on the right above). The provided version can recognize the colors white, yellow and green and the digits 1 to 8. The software can further detect bees that enter or leave cavities (as in the image on the right below) and can therefore detect which bee has entered or left a cavity at what point in time. From these detected events further measurements can be extracted such as assigning marked females to the cavity they are nesting in. Additionally, flight durations and the number of cavities probed until a bee has found its own "correct" cavity are detected by the software.

4.2 Inputs

4.2.1 Input videos

The input videos should contain a nesting unit with cavities as in the above image. The cavities will be numbered A1, A2,...,Ax for the first row, B1,B2,...,Bx for the second row and so on.

Important: The camera should not be moved during the whole video! The cavities of the nesting unit must be in the same position during the whole video.

The input videos should be in MP4 format and the aspect ratio should be 16:9. For example 3860x2160 (4K).

The software is much more efficient if you provide more than one input video. For example, if you want to analyze three videos, do not start the software three times but choose all three videos as inputs and run the software for all three of them.

4.2.2 Results Folder

Choose an empty folder as the results folder. The software will create a new folder for each input video in the results folder and save all final and intermediate results to this folder. The result folders are labelled according to the file names of input videos. Input videos should therefore labelled with unique names.

4.2.3 Config File

Optionally, a *config* file can be provided as a third input. Inside the *config* file, the below parameters can be altered. If no *config* file is provided, the software will use the default values visible in the image below.

```python
'''
Choose the video size of the visualizations that are generated by the software.
'''
visualization_video_size=(2048,1152)


'''
Define the number of worker threads. It defines how many videos are processed in
parallel at most.
Note that the bottleneck of the Software is the GPU. There will be no benefit in
choosing a too high num_worker_threads value. 2-6 worker threads should be sufficient
to keep the GPU fully utilized.
'''
num_worker_threads = 6


'''
A bee will only be tracked over time if it is not further apart than
(max_tracking_distance_factor*average_hole_height) in two consecutive frames
'''
max_tracking_distance_factor = 1.4


'''
A start of a flight is only counted if the area of the bee detection bounding
box is smaller than hole_area_factor*average_hole_area.

'''
hole_area_factor = 1.2


'''
A bee is only considered for the evaluation if it is being tracked over at least
min_consecutive_frames_to_track_bee frames.
'''
min_consecutive_frames_to_track_bee = 3


'''
The min_nest_time denotes the minimal time, a bee has to stay inside a hole to qualify
for residency. (in milliseconds)
'''
min_nest_time = 40000


'''
The min_fly_time denotes the minimal time a bee has to be in flight after leaving
a hole to qualify for residency. (in milliseconds)
'''
min_fly_time = 40000


'''
The following variables define the trained deep learning model paths. Make sure
to only use forward slashes and NO BACKSLASHES in your paths.
'''
bee_model_path = "bee_model"
hole_model_path = "hole_model"
color_model_path = "color_model.h5"
number_model_path = "number_model.h5"
```

4.3 Outputs

The software will create a new subfolder within the results folder for each input video. Inside each such subfolder the following final outputs can be found:

1. **all_events_unfiltered.csv:** Inside this csv file, all detected events are listed in the following format: TIME, BEE_ID, MOVEMENT (enter or leave), CAVITY_ID. Each event has a timestamp and defines which bee has entered or left which cavity (see example output on the right). This list is completely unfiltered and may contain errors.

11	0:00:38.160000,yellow8,Enter,E3
12	0:00:38.400000,yellow3,Enter,D10
13	0:00:38.640000,yellow3,Leave,D10
14	0:00:41.080000,green5,Enter,E13
15	0:00:43.560000,yellow2,Leave,D7
16	0:00:44.560000,yellow3,Enter,D8

2. **error_corrected_events.csv:** This csv file contains all events that remain after error deletion. The data is listed in the following format: TIME, BEE_ID, MOVEMENT (enter or leave), CAVITY ID, USED (event used to create measures in output files are listed as residential movements).

3. **address_book.csv:** Inside this csv file, all detected “addresses” are listed in the following format: BEE_ID, CAVITY_ID, EVIDENCE. Each bee is assigned to one (or more) cavities. The evidence indicates the number of occurrences of a bee entering the cavity.

4. **nest_recognition.csv:** Inside this csv file, the number of wrong cavities a female enters before finding its nest is listed in the following format: TIME, BEE_ID, CAVITIES.

5. **flight_list.csv:** Inside this csv file, the flights are listed in the following format: TIME, BEE_ID, DURATION. Note that flights might be missing in this file due to the strict filtering of events in case of mispredictions by the deep learning models. (Read the “Extracting Measurements” section for more details).

6. **visualization.mp4:** If you have activated the “Visualize Results” option, a video file called visualization.mp4 will be created with all detected events visualized.

In addition to the final outputs, some intermediate results are stored. Read the “Inner Workings” section for further information on these intermediate results.

4.4 Start / Pause / Resume

Once you have defined your inputs, you can start the computations by clicking on the “Start” button. You can pause the software anytime by clicking the “Pause” button. It can take a while (up to a few minutes) until the software is stopped. Please do not close the window or shut down your computer during this time. Otherwise it might not be possible to resume the computations. By clicking on “Start” again, the software can be resumed from the position it was paused.

Once the software has completed its computations it can be rerun with different parameters in the *config* file. This will not rerun all computations but only part of it. Therefore the computations will be much faster this time!

4.5 Inner Workings

4.5.1 Detecting Bees

The first and most time consuming step of the software is to detect all bees in all frames of the video. The Faster RCNN Object Detection architecture with an image size of 1024x576 is used for this step. The software detects two types of classes: *bee* and *bee flying*.

The software will skip 12 frames (approximately 0.5 s) if no bees are detected. This is to speed up the computations in case of no bees being present in the video.

4.5.2 Detecting Cavities

The second step is to detect the positions of all cavities in the video. This is done with the Faster RCNN Object Detection architecture with an image size of 1024x576 as well. The cavities are only detected in a few frames. Therefore this step does not consume a lot of time.

A visualization of the detected cavities is stored to the folder *frames_without_bees* inside the output folder as *detected_holes.jpg* (see image below). If cavity positions are detected with any error, these can be corrected as described in the Troubleshooting section.

For the detection of cavity positions the software requires cavities that are arranged in horizontal rows. The camera should not be moved during the whole video! The cavities of the nesting unit must be in the same position during the whole video.

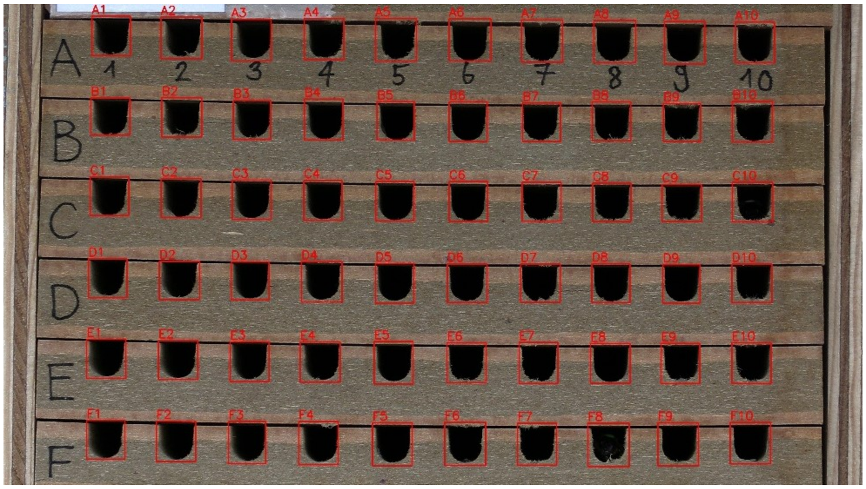

4.5.3 Detecting Colors

The third step is to detect all colored IDs that are glued to the thorax of the bees. For this step, the images of the detected bees in step 1 are analyzed with the YOLOv3 Object Detection architecture. The YOLOv3 network will output the coordinates of the bounding box and the color of the ID as white, yellow or green (see image below).

In order to speed up this computation, the bee images of flights that do not end or start in a cavity are not analyzed.

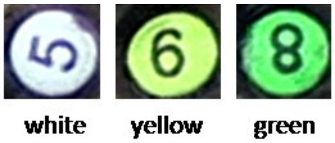

4.5.4 Detecting Numbers

The fourth step is to recognize the numbers on the detected IDs from the previous step. A custom convolutional neural image classification network with 3 convolution layers and an input image size of 50x50 pixels is used. It has 9 possible output classes: the digits 1-8 and the *indistinctive* class. If the number is blurry, cut off or at a bad angle, the network will predict *indistinctive*. Due to its small size, this network can be executed extremely fast on a huge amount of detected number tags.

4.5.5 Event Detection

Having applied these four neural networks to the input video, the positions of all bees (along with the color-digit combination in the ID, if present) in each frame and the positions of all cavities are known to the software. The next step is to extract the events from this data. There are two possible events: entering or leaving a cavity.

The first step to reach this goal is to implement an object tracking algorithm. It tracks bees over multiple frames and assigns them an ID. A customized centroid tracking algorithm is used for this task. For a bee to be tracked over multiple frames, the centers of the bounding boxes within two consecutive frames are required to be within a certain *max_distance* (definable in the *config* file, default is 1.4 x *average_hole_height*). If this requirement is fulfilled the closest detections within two consecutive frames are assigned the same ID. However, if two detections of flying bees are close by (within 2 *x max_distance*), the bees are not tracked because the risk of ID swapping is high. Flying bees are only tracked if there are no other bees within 2 x *max_distance*).

After individual bees got assigned an ID they can be tracked over multiple frames. The color and digit of the ID can therefore be detected with very high accuracy as multiple frames can be used for identification.

Finally, the software can detect the events (entering and leaving a cavity). To do so, the starting and end points of all flights (tracked bee IDs) are analyzed. If the starting point of a flight is within the bounding box of a cavity and the detection is *bee* (and not *bee flying*), the cavity at that location is taken as the starting point of this flight. The detection of flight end points works analogous. There is a parameter in the *config* file which defines the maximal size of a bee detection to be considered as a starting or end point. Default is 1.2 x *average_hole_area*.

4.5.6 Extracting Measurements

To extract measurements a csv file with the detected events (*all_events_unfiltered.csv*) is used as input. Every row contains the movement of a bee. The array (data) is organized as following:

1. Time stamp (MM:SS)

2. Bee ID (color-digit combination)
3. Movement (*Enter* or *Leave*)
4. Cavity ID

The data is ordered by the time stamp. First, a cavity-based error correction is made by sorting the data by cavity ID. The movements are checked iteratively for repeated *Enter* or *Leave*. Such errors are corrected by *correct_missing_movement*.

correct_missing_movement complements unpaired *Enter* or *Leave* with a placeholder event. The time stamp of placeholder events are set one millisecond after the referring *Enter* or one millisecond before the referring *Leave*. Placeholder events are labelled as *Missing Enter/Leave*.

After the cavity-based error correction a second correction step is carried out which is based on bee IDs. For this purpose the data is ordered primarily by time stamp and secondarily by the bee ID. Unpaired events are again corrected by *correct_missing_movement*as as described above.

Based on this error-corrected data the measurements are extracted. First, nesting females are assigned to the cavities they are nesting in. A bee only gets assigned to a cavity, if it stays inside the cavity for *min_nest_time* after entering it and does not enter any other cavity within *min_fly_time* after leaving. The default values for both *min_nest_time* and *min_fly_time* are 40 s and can be changed in the *config* file. The implementation of this step is done in *check_resident* by iterating through the data.

check_resident always start with an *Ente*r event and then calculates the two relevant times (time inside and outside cavities) which are compared to the set time thresholds. Considered events that fulfill the time criteria are saved in *residential_movements* and the further processing by *take_measurement* gets activated. Placeholder events are not considered for the calculations.

take_measurements calculates two values: *drunkenness* und *collection time*. The *drunkenness* is the number of cavities a bee probes until it finds the one it was assigned to (and is allegedly nesting in) while the *collection time* is calculated as the duration from leaving till returning to the nest.

Finally, three output csv files with the following measurements are created:

address_book mit der bereinigten Liste der benisteten Löcher (und ihren Bewohnern)

- *address_book* which contains the list of females and the cavities they are nesting in
- *nest_recognition* which lists the number of cavites a female probed until it found ist nest
- *flight_book* which liests flight durations

4.6 Troubleshooting

Not all cavities in the nesting unit are detected by the software:

It is highly advised to have a look at the visualization of the detected cavities before using any results. You can find the visualization within the *frames_without_bees* folder (inside the output folder of each video) as *detected_holes.jpg*. If the cavities are not detected correctly, you can edit them by hand as follows:

1. Start the *LabelImg* application. There is a *LabelImg.exe* file within the *BeeTracker.zip* file containing the software.

2. Open the *detected_holes.jpg* image with the *LabelImg* application and edit the cavity annotations within the *detected_holes.xml* file.
3. Delete the *detected_holes.pkl* file within the results folder of the video in question. Also delete the *detected_colors.pkl* and the *detected_numbers.pkl* within the same folder.
4. Restart the software. It will now take your edited cavity annotations to detect all events.

5 Retraining Computer Vision Models

The used models can be retrained with new or additional data. This might be necessary if more colors should be detected or a different looking bee species. To retrain a network, some Python programming knowledge is beneficial.

To be able to retrain the networks, you have to install the conda environment with all necessary packages to train the networks. To do so, carry out the following steps:

1. Download Anaconda with Python 3.7 from https://www.anaconda.com/distribution/ and install it.

2. Open the Anaconda Prompt Program, change to the *Code Repository* folder within the extracted zip file by running the following command:

   cd /path/to/Code Repository/on/your/computer

3. Then type the following command to install the conda environment:

   conda env create -f environment.yml

   This will install all python dependencies including the Tensorflow library with GPU support.

Once you have set up your conda environment, you are ready to retrain the models.

5.1 Retrain Bee Model

5.1.1 Preparing Training Data

In order to train a neural network to detect one or multiple classes of objects, it has to be trained with a large amount of training data. Follow the steps below to prepare your data.

1. The first step is to collect a large amount of images containing the objects you want to detect. Place all these images into one (or multiple) folders.
2. Open the *LabelImg* program inside the extracted zip folder.
3. Use the *LabelImg* program to annotate all the objects within your images. To do so,
   - Click on “Open Dir” and select the folder containing the images.

- Click on “Change Save Dir” and select the same folder (containing the images)
- By clicking “w”, annotate all objects (e.g. *bee flying*) within the first image.
- Make sure to save your annotations by clicking on “Save”.
- Do the same for the other images.

To retrain the bee model, all bees have to be annotated either as *be*e or *bee flying* in *LabelImg*.

5.1.2 Training the network

Open the *image_preprocessing_bees.py* script in a text editor. At the bottom define all parameters, including the *input_folders*. This is an array of folder paths containing the training data. Make sure to also define the *test_splits* and *validation_splits* array variables as well as a *project_folder*. The *project_folder* should be an empty folder where all training results will be saved to. Then run the script. It will prepare everything for training including downloading a pretrained model.

Once the *image_preprocessing_bees.py* script has finished, open the *train_with_validation.py* script in a text editor. Edit the *project_folder* to match the *project_folder* used in the *image_preprocessing_bees.py* file and execute it. The training can take up to 24 hours, however mostly a well performing model has already been found after a few hours.

Finally, open the *my_export_inference_graph.py* file and edit the *project_folder* variable again, then execute it. The trained model will be saved to the *trained_inference_graphs/ output_inference_graph_v1.pb* subdirectory of the project folder. You can specify this path in the *config* file to use it for the predictions within the Bee Tracker Software.

5.2 Retrain Cavity Model

Do exactly the same as for the bee model training, except use the *image_preprocessing_holes.py* file instead of the *image_preprocessing_bees.py* file. Annotate the cavities by naming them *hole* in the training data.

5.3 Retrain Color Model

The first two steps are the same as for the bee model training. Annotate your images with the *LabelImg* application. Draw the bounding boxes around the colored number tags and label it with the color (not the number). Then modify and run the *image_preprocessing_colors.py* file.

5.3 Retrain Digits Model

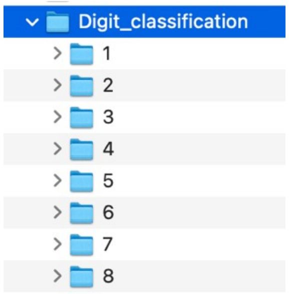

To retrain the digits model, the training data has to be prepared in the following structure: There must be a folder containing 8 subfolders named from 1 to 8 (as in the image on the right). Each subfolder contains a list of images of the corresponding digit. All images will be resized to 50x50 pixel images for the network training.

Finally, use the train command in the *train_digits.py* file to train the network. You must provide the input folder (Digit_classification in the example screenshot) and an output location for the trained model.

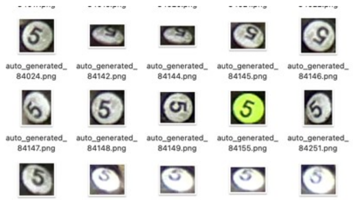

Supplement: Supplementary file 1 — Supplementary Material [file ECE3-12-e8575-s001.pdf]
